# Genetic and environmental influences on the developmental trajectory of callous‐unemotional traits from childhood to adolescence

**DOI:** 10.1111/jcpp.13259

**Published:** 2020-05-17

**Authors:** Yusuke Takahashi, Christopher R. Pease, Jean‐Baptiste Pingault, Essi Viding

**Affiliations:** ^1^ The Hakubi Center for Advanced Research, and Division of Cognitive Psychology in Education Graduate School of Education Kyoto University Kyoto Japan; ^2^ Department of Clinical, Educational and Health Psychology Division of Psychology and Language Sciences University College London London UK; ^3^ Social, Genetic and Developmental Psychiatry Centre King's College London London UK

**Keywords:** Callous–unemotional traits, trajectory, genetic and environmental aetiology, latent growth model, twin study

## Abstract

**Background:**

This study examined the genetic and environmental influences underlying baseline level and developmental course of callous–unemotional (CU) traits across childhood and adolescence.

**Methods:**

The data on 8,958 twin pairs (3,108 MZ twin pairs and 5,850 DZ twin pairs) from the Twins Early Development Study were analysed. CU traits were assessed at ages 7, 9, 12 and 16 by mothers and analysed using a biometric latent growth model.

**Results:**

Individual differences in the baseline level of CU traits were highly heritable (76.5%), while the heritability of the developmental course of CU traits was moderate (43.6%). The genetic influences on baseline level and developmental course of CU traits were mostly nonoverlapping. Nonshared environment made a modest contribution to the baseline level of CU traits (21.7%). Nonshared environmental influences on the developmental course of CU traits were moderate (43.2%), with nearly half of them being the same as those influencing the baseline level and just over half being specific. Shared environmental effects did not contribute to systematic change across childhood and adolescence but were rather age‐specific.

**Conclusions:**

Our findings demonstrate that rather than only being conceptualized as factors of stability, genes also play a dynamic role in explaining systematic change in CU traits. Genetic effects for the initial risk and subsequent development of CU traits are not the same. In addition to genetic factors, nonshared environmental influences play an important role in explaining why some children will increase or maintain their CU traits over time, whereas other will desist. New genetic and environmental influences with age suggest that repeated, age‐tailored interventions may be required throughout development to make a lasting difference in the presentation of CU traits and associated outcomes.

## Introduction

Callous‐unemotional (CU) traits – including diminished ability to feel guilt and reduced concern for other people's feelings – characterize a subset of children at risk of developing persistent antisocial behaviour (Pardini & Frick, 2013; Viding & Kimonis, 2018). CU traits have received interest as a specifier for conduct disorder diagnosis in the DSM‐5 (termed ‘limited prosocial emotions’; Frick, Ray, Thornton, & Kahn, 2014), but also in their own right as an indicator of risky outcomes besides conduct problems, such as substance use, criminal offending and childhood psychopathology (Anderson, Zheng, & McMahon, 2018; Kahn, Byrd, & Pardini, 2013; Moran et al., 2009).

Studies investigating the origins of CU traits using twin data have proliferated in the past 15 years (Viding & McCrory, 2012, 2018). Collectively, these studies indicate that a significant amount of variance in CU traits is explained by additive genetic factors. Nonshared (i.e. individual‐specific) environmental factors also reliably play a role, and few studies report small contribution from shared environmental factors (Bezdjian, Raine, Baker, & Lynam, 2011; Fontaine, Rijsdijk, McCrory, & Viding, 2010; Larsson, Andershed, & Lichtenstein, 2006; Viding, Frick, & Plomin, 2007). Specifically, reviews of the literature (Moore, Blair, Hettema, & Roberson‐Nay, 2019; Viding & McCrory, 2012) indicate that between 36% and 78% of the variance in CU traits in children and young people is due to genetic factors, suggesting that some individuals have greater genetic vulnerability to developing CU traits than their peers. The findings from twin studies accord with important adoption data showing that children whose biological parents display antisocial behaviours are at a higher risk of developing CU traits (Waller et al., 2016). In other words, there is a genetic vulnerability to expressing CU traits and this can manifest even in children who are adopted at or shortly after birth. The quality of (adoptive) parenting such as parental warmth and consistent reinforcement buffer the effects of heritable risk for CU traits, and this unequivocally shows the importance of environmental factors (Hyde et al., 2016; Waller et al., 2016). The adoption studies have thus been important in complementing the twin study evidence base and demonstrating the importance of both genetic risk and environmental influences in emergence of CU traits.

Phenotypic studies suggest that at the population level, CU traits are at least as stable as other predispositional traits across childhood, adolescence and early adulthood (Barry, Barry, Deming, & Lochman, 2008; Frick, Kimonis, Dandreaux, & Farell, 2003; Lynam, Charnigo, Moffitt, Raine, Loeber, & Stouthamer‐Loeber, 2009; Pardini & Loeber, 2008; Viding & Kimonis, 2018). However, we also know that there can be remarkable differences between individuals in the developmental trajectories of their CU traits (Fontaine et al., 2010). The relative importance of genetic and environmental factors in explaining development of CU traits has received limited attention to date, with only a handful of longitudinal twin studies focusing on these traits in childhood or adolescence/early adulthood. For instance, Flom and Saudino (2017) measured CU traits at ages two and three years and demonstrated a genetic correlation of .63 between these time points. This means that there is a substantial overlap between those genetic factors that predispose to CU presentation at toddler and preschool ages. Forsman, Lichtenstein, Andershed, and Larsson (2008) showed that genetic factors (both those shared with other aspects of psychopathic personality and those unique to CU traits) were largely shared between CU traits measured at ages 16 and 19 years. There was also some, although lesser, overlap between nonshared environmental factors that contributed to variation in CU traits at ages 16 and 19. Fontaine et al. (2010) conducted person‐centred analyses on CU traits between ages 7, 9 and 12 and demonstrated that baseline levels of CU traits (i.e. high or low) were strongly to moderately heritable. This study also demonstrated substantial genetic influences on increasing and decreasing developmental trajectories of CU traits, suggesting that some of the systematic changes seen in these traits may be driven by genetic, not just environmental, influences. However, person‐centred trajectory models are not able to tell us whether genetic and environmental influences on the baseline level and the developmental course of CU traits are the same or different.

Prior work on other phenotypes (e.g. conduct problems and ADHD; Pingault, Rijsdijk, Zheng, Plomin, & Viding, 2015; Pingault, Viding, Galéra, Greven, Zheng, Plomin, & Rijsdijk, 2015) has documented that in addition to genetic continuity, genetic effects are developmentally dynamic, which means they vary over time – a phenomenon dubbed ‘genetic innovation’. More specifically, genetic innovation indicates novel heritable effects that become apparent over the course of development with previously inactive genes coming online. Genetic innovation may result from maturational processes (e.g. long‐term neural maturation or neural rewiring around puberty) or reflect gene–environment or gene–gene interactions (a gene coming into play following exposure to a new environment or a newly activated gene). Similarly, genetic factors that were not expressed at an early age can start influencing CU traits at a later age, pointing towards the importance of genetic factors in age‐to‐age change. Although existing longitudinal studies show genetic influences on age‐to‐age change in CU traits (Flom & Saudino, 2017; Fontaine et al., 2010; Forsman et al., 2008), they do not directly address the role of genes in the developmental course (i.e. systematic changes occurring with age, such as linear increases or decreases) of CU traits. The same considerations apply for ‘environmental innovation’. A better understanding of the degree to which new genetic and environmental influences may arise across development will provide important directions for the future studies that measure specific genotypes or environmental inputs, and can yield insights for conceptualizing intervention needs. For example, the findings may be helpful in guiding molecular genetic investigations, as they can help arbitrate whether different genetic effects would be expected at different time points. They can also guide intervention efforts, as new genetic and environmental effects coming ‘online’ during development suggest a need to repeat and adapt intervention approaches for the most vulnerable.

Thus, in this study we sought to test the following research questions: Are the same genetic factors largely responsible for individual differences in the baseline level of CU traits and their continuity over time, whereas environmentally driven socialization processes explain individual differences in the developmental course? Or might genetically dependent developmental processes also impact the expression of CU traits over time (i.e. genes explain not only continuity but also systematic change)? The respective role of genes and the environment in explaining the developmental course of CU traits can be addressed by (a) using a latent growth model to explicitly examine the baseline level (i.e. a latent factor based on all measurement points, which estimates the intercept) and the developmental course (i.e. systematic change such as a latent factor representing the linear slope) of CU traits; and (b) incorporating genetic and environmental influences on the latent structure (i.e. again, the intercept and the linear slope; Pingault, Rijsdijk et al., 2015; Pingault, Viding et al., 2015). Such a model provides a direct estimate of the genetic and environmental contributions to the developmental course of CU traits; it also enables the distinction between contributions specific to the developmental course and those shared with the baseline level.

Using this approach, Henry et al. (2018) analysed teacher ratings of CU traits in a sample of 622 twin pairs aged 7, 9, 10 and 12. They reported that genetic factors accounted for most of the variance in the baseline of CU traits (i.e. 89%). Sadly, they were unable to conduct genetically informative analysis of the systematic change over time (as reflected in the variance of the slope parameter) because of the absence of phenotypic change in their sample. Cholesky decomposition analysis showed that genetic factors at age 7 maintained influence throughout the remaining time points, while genetic innovation also occurred at later ages. These findings are in line with earlier work in adolescence (Forsman et al., 2008) and leave open the possibility of distinct genetic effects on the intercept and slope of CU traits. Henry et al. (2018) also showed that nonshared environmental influences were mostly age‐specific and did not detect shared environmental influences on CU traits in their sample.

Taken together, the extant studies collectively suggest that genetic influences are important for accounting for both initial risk of developing CU traits and their stability. What remains less clear, however, is *the degree to which* genetic influences that impact initial risk for versus development of CU traits across childhood and adolescence differ or overlap. The prior studies have typically focused on relatively short time periods in early childhood, preteen or late teen period. No prior study spans an age range that captures the period from middle childhood to adolescence, an important neurodevelopmental window where psychiatric vulnerability often consolidates (Foulkes & Blakemore, 2018). Throughout childhood and adolescence (more specifically, at ages 6–21; see Frick et al., 2014), elevated CU traits have been both concurrently and longitudinally linked to delinquency, aggressive behaviours and poorer response to treatment. Additionally, although CU traits have been shown to be relatively stable throughout childhood and adolescence, few longitudinal studies have reported on either decreases or variable trajectories of CU traits over time (Blonigen, Hicks, Krueger, Patrick, & Iacono, 2006; Fontaine et al., 2010). Given the negative longitudinal outcomes associated with elevated levels of CU traits, aetiologically informative evidence in adolescence (especially, after the (junior) high school years) is important for understanding underlying mechanisms of individual differences in CU traits.

Here, we report analyses on a large twin sample assessed at ages 7, 9, 12 and 16, to (a) examine the genetic and environmental influences underlying developmental course of CU traits and (b) verify whether these developmental influences were independent from or shared with those influencing baseline CU traits.

## Methods

### Participants

Participants are part of the Twins Early Development Study (TEDS), a longitudinal population‐based study of twin pairs in England and Wales, recruited at birth between 1 January 1994 and 31 December 1996 (Haworth, Davis, & Plomin, 2013). The present study sample included a total of 8,958 twin pairs (3,108 MZ twin pairs and 5,850 DZ twin pairs) from whom callous–unemotional trait scores rated by twins' mothers were available in at least one of four assessments between ages 7 and 16 years [i.e. 7.07 years (*SD* = 0.25 years), 9.02 (0.29), 11.31 (0.72), 16.32 years (0.68)]. Twins with severe medical problems or severe birth complications were also excluded from the sample. Table 1 shows the number of complete MZ and DZ twin pairs used in this study and intraclass correlations at each age (see Appendix S1 for information on zygosity determination). Opposite‐sex twin pairs were excluded from all analyses in this study, to prevent sex differences from inflating estimates of genetic effects.

**Table 1:** Descriptive statistics for CU traits at each age, phenotypic correlations and intraclass correlations

Age (years)	*M*	*SD*	Skewness	Kurtosis	*ω*	Phenotypic correlations [with 95% CI]					*N* of completed pairs	*N* of MZ pairs	*N* of DZ pairs	Intraclass correlations [with 95% CI]	
						Age	Sex	7 years	9 years	12 years				MZ	DZ
7	3.07	2.04	.71	.73	.75	−.05 [−.08, −.03]	.14 [.12, .17]	‒			7,716	2,726	4,990	.65 [.63, .68]	.35 [.32, .37]
9	2.70	1.85	.72	.49	.81	−.01 [−.05, .02]	.17 [.13, 20]	.41 [.38, .44]	‒		3,383	1,238	2,145	.80 [.78, .82]	.56 [.53, .59]
12	2.33	1.84	1.07	1.96	.80	−.02 [−.05, .00]	.16 [.13, .18]	.36 [.34, .39]	.43 [.40, .46]	‒	5,841	2,105	3,736	.76 [.74, .78]	.47 [.45, .50]
16	2.61	2.16	.96	.94	.82	−.04 [−.06, −.01]	.17 [.15, .20]	.31 [.28, .33]	.34 [.31, .38]	.41 [.38, .44]	5,089	1,818	3,271	.82 [.81, .84]	.54 [.52, .56]

95% confidence intervals are reported in brackets.

### Ethical considerations

Ethical authorization, including authorization to work with children, was given by the Joint South London and Maudsley and the Institute of Psychiatry Research Ethics Committee. Participants' parents were given a letter describing the general purpose of the study, and written informed consent was obtained.

### Measurement of CU traits

CU traits at 7, 9, 12 and 16 years of age were assessed by a seven‐item scale rated by mothers, as used in previous heritability analyses of CU in TEDS (Viding et al., 2007). CU scores were composed of four Strengths and Difficulties Questionnaire (SDQ; Goodman, 1997) items selected to reflect CU [i.e. ‘Considerate of other people's feelings (reverse‐scored)’, ‘Helpful if someone hurt (reverse‐scored)’, ‘Have at least one good friend (reverse‐scored)’ and ‘Kind to younger children (reverse‐scored)’] and three Antisocial Process Screening Device (APSD; Frick & Hare, 2001) CU subscale items [i.e. ‘Does not show feelings or emotions’ ‘Guilty when does something wrong (reverse‐scored)’ and ‘Concerned to do well (reverse‐scored)’]. Each item was rated on a 3‐point Likert scale (0 = *Not true*, 1 = *Somewhat true* and 2 = *Certainly true*). The CU score at age 16 was also computed from seven items. Items were the same four SDQ items, and three items from the Inventory of Callous–Unemotional Traits (ICU; Frick, 2003) with the same item content than the APSD CU subscale, but rated on a 4‐point Likert scale ranging from 0 (*Not at all true*) to 3 (*Definitely true*). When creating the composite score for age 16, to adjust score range of the ICU to the APSD, we applied a linear transformation (Colman, Norris, & Preston, 1997) to the ICU's scaling changing it from 0 to 3, to 0 to 2 to match the APSD. These three item scores were multiplied by two thirds before creating the composite score in CU traits for age 16. All scores were regressed on age and sex prior to analyses in this study. A more detailed description of CU trait measurement can be found in Appendix S2.

### Statistical analysis

#### Phenotypic analyses

A latent growth model was fitted to examine the developmental trajectory of CU traits between ages 7 and 16 years. First of all, a phenotypic latent growth model was fitted to the data to determine the baseline level (i.e. intercept) and to test whether a linear slope was sufficient to account for the observed systematic change in CU. Because our longitudinal data have a small number of measurement points (i.e. four), we did not fit a quadratic term to avoid overfitting. Intercept factor loadings were fixed to one for all waves to capture baseline levels. Slope factor loadings were fixed to 0 for the first wave and to 0.2, 0.5 and 0.9 for the second, third and fourth waves in order to reflect differences at assessment ages (i.e. difference in years from the starting point divided by 10). Time‐specific residuals, that is variance at each time point not explained by the growth factors, were also estimated.

#### Genetic analyses

We first tested univariate genetic models of CU traits at 7, 9, 12 and 16 years. Univariate genetic models can give us estimates of relative genetic and environmental contributions on individual differences in CU traits at each age.

Based on covariances within MZ and DZ twin pairs, univariate models decompose the phenotypic variance into additive genetic (A), common environmental (C) and nonshared environmental (and measurement error) (E) factors.

Multivariate genetic analyses were then carried out in order to investigate the aetiology of the development of CU traits over this developmental period. The first multivariate genetic analysis was a Cholesky decomposition of the phenotypic variance at all four waves. Applying a Cholesky decomposition to longitudinal data allows us to specify genetic and environmental variances that are time‐specific and/or shared across the waves. The second multivariate genetic model was a latent growth model delineating two latent factors: the intercept, which refers to the baseline level of CU traits, and the linear slope, which refers to the rate of increase or decrease with age (or systematic change) and models the developmental course of CU traits. The latent growth model also includes the covariance between the intercept and the slope. This model enables the estimation of how much of the genetic and environmental influences on the developmental course of CU traits (i.e. linear slope) are shared with influences on baseline levels (i.e. intercept). Each wave‐specific residual was also decomposed into ACE factors.

Full information maximum likelihood was used to deal with missing data. Because the CU trait scores were positively skewed (Table 1) as expected, we used a robust maximum likelihood estimator to allow the use of all available data while remaining robust to non‐normality. To evaluate the goodness of fit of the relevant model, we considered three different criteria: the robust version of the comparative fit index (robust CFI), the robust version of the root mean square error of approximation (robust RMSEA) and the standardized root mean square residual (SRMR). According to Hu and Bentler (1999), values of CFI > 0.95, RMSEA < .060 and SRMR < .080 are indicative of good fit. The psychometric package *psych* 1.8.10 (Revelle, 2018) and the structural equation modelling package *lavaan* 0.6‐3 (Rosseel, 2012) implemented within R software version 3.5.1 (R Core Team, 2017) were used for phenotypic and biometric models.

## Results

### Phenotypic analyses

**Figure 1:**
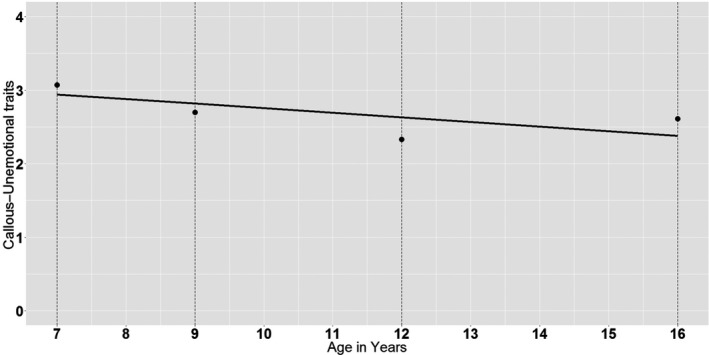
Developmental trajectory of CU traits from ages 7 to 16. Observed mean values of CU (dots in black) and model fitted linear decrease (a line in black) are represented

Table 1 shows the descriptive statistics, reliability coefficients, correlations between waves, number of twin pairs who participated at each wave and intraclass correlations. A linear decrease in mean levels of CU traits across childhood and adolescence was observed, although a slight increase was found between 12 and 16 years of age. Ordinal omega coefficients (McDonald, 1999) were all acceptable. Spearman's rank‐order correlations were used to estimate the stability coefficients among the CU traits (see Table 1). The stability coefficients were moderate ranging from .31 between 7 and 16 years of age to .43 between 9 and 12 years of age (all *p*s < .01). Thus, at a rank‐order level, the CU traits were found to be fairly stable across time. Also, small correlations with age and sex were found. Original scores and scores residualized for age and sex were highly correlated (i.e. all *r*s > .98). As shown in Table 1, the intraclass correlations were greater for the MZ than the DZ twin pairs at each wave, suggesting significant genetic influences. Next, at the phenotypic level, the latent linear growth model was tested and fitted adequately to the data (*χ* ^2^ = 671.79, *df* = 65, *p* < .01, robust CFI = .954, robust RMSEA = .041, SRMR = .055). The mean and variance for intercept (I) significantly differed from zero (*M_I_* = 2.97, *SE* = .02, *p* < .01; *σ^2^_I_* = 1.83, *SE* = .07, *p* < .01). The mean and variance for the linear slope (S) also significantly differed from zero (*M_S_* = −.66, *SE* = .03, *p* < .01; *σ^2^_S_* = 1.27, *SE* = .19, *p* < .01). The score of CU traits gradually decreased from a score of 3.07 at age 7 years to a score of 2.61 at age 16 years, and observed mean values of CU (dots in black) and model fitted linear decrease (a line in black) are represented in Figure 1.

### Genetic analyses

#### Univariate genetic models

Univariate genetic models were tested on the CU trait scores at each wave (Table 2). Table 2 includes the estimates and 95% confidence intervals for A, C and E as found in the full ACE model. As expected from the intraclass correlations shown in Table 1, the observed variances in the CU traits were moderately heritable. Half (or more) of the total variance was explained by genetic factors in the full ACE model at all waves. Also, significant shared environmental effects ranging from .20 to .31 were detected except at 7 years of age.

**Table 2:** Univariate variance estimates [with 95% CIs] of additive genetic (*a* ^2^), shared environmental (*c* ^2^) and nonshared environmental (*e* ^2^) contributions to CU traits at each age

Age (years)	Parameter estimates [with 95% CI]			CFI	RMSEA	SRMR
	*a* ^2^	*c* ^2^	*e* ^2^			
7	.655 [.626, .684]	.015 [−.010, .040]	.330 [.318, .342]	.993	.025	.035
9	.491 [.458, .524]	.314 [.289, .339]	.195 [.185, .205]	1.000	.000	.020
12	.555 [.526, .584]	.198 [.174, .222]	.247 [.237, .257]	.996	.026	.027
16	.583 [.556, .610]	.245 [.223, .267]	.172 [.164, .180]	.999	.016	.025

95% confidence intervals are reported in brackets.

#### Cholesky decomposition

We next fitted a standard Cholesky decomposition as is commonly used on longitudinal twin data (Table 3). The influence of additive genetic factors was consistent at all ages, explaining over 50% of the total variance at each age. From a longitudinal perspective, there was evidence for both genetic continuity and genetic innovation. For instance, genetic factors explaining CU at age 7 years still explained 14% of the total variance at age 16 years, and at the same time, 36% of the variance at age 16 years was independent of genetic influences at all previous ages. Shared environmental influences were small at several waves and may contribute to the stability over one or two ages. Nonshared environmental influences were moderate and largely age‐specific, indicating that there was no clear evidence on any environmental continuity across ages. Although this lack of evidence for environmental continuity may reflect the role of the environment in change, we should keep in mind that nonshared environmental influences include measurement error.

**Table 3:** Cholesky decomposition of additive genetic, shared environmental and nonshared environmental influences for CU trait score from ages 7 to 16 [with 95% bootstrap CIs]

	Proportion with 95% CI				
	Age 7	Age 9	Age 12	Age 16	Total
Additive genetic effect (A)					
Age 7	.671 [.647, .690]				.671 [.647, .690]
Age 9	.226 [.139, .285]	.287 [.189, .370]			.513 [.416, .602]
Age 12	.167 [.108, .214]	.096 [.004, .198]	.307 [.218, .398]		.571 [.463, .669]
Age 16	.135 [.102, .193]	.023 [.002, .088]	.067 [.014, .134]	.364 [.306, .457]	.589 [.525, .676]
Shared environmental effect (C)					
Age 7	.001 [−.062, .063]				.001 [−.062, .063]
Age 9	.205 [.093, .355]	.092 [−.336, .271]			.297 [.209, .378]
Age 12	.020 [−.024, .206]	.158 [.108, .221]	.005 [−.228, .204]		.184 [.090, .268]
Age 16	.004 [−.134, .282]	.055 [.000, .265]	.103 [−.046, .236]	.080 [−.165, .224]	.241 [.170, .308]
Nonshared environmental effect (E)					
Age 7	.329 [.303, .349]				.329 [.303, .349]
Age 9	.017 [.011, .027]	.174 [.146, .202]			.191 [.168, .223]
Age 12	.014 [.009, .023]	.024 [.011, .035]	.206 [.182, .239]		.245 [.218, .275]
Age 16	.002 [.001, .005]	.002 [.000, .006]	.009 [.004, .018]	.157 [.135, .182]	.170 [.146, .194]

95% confidence intervals obtained by bootstrapping are reported in brackets.

#### Latent growth model

**Figure 2:**
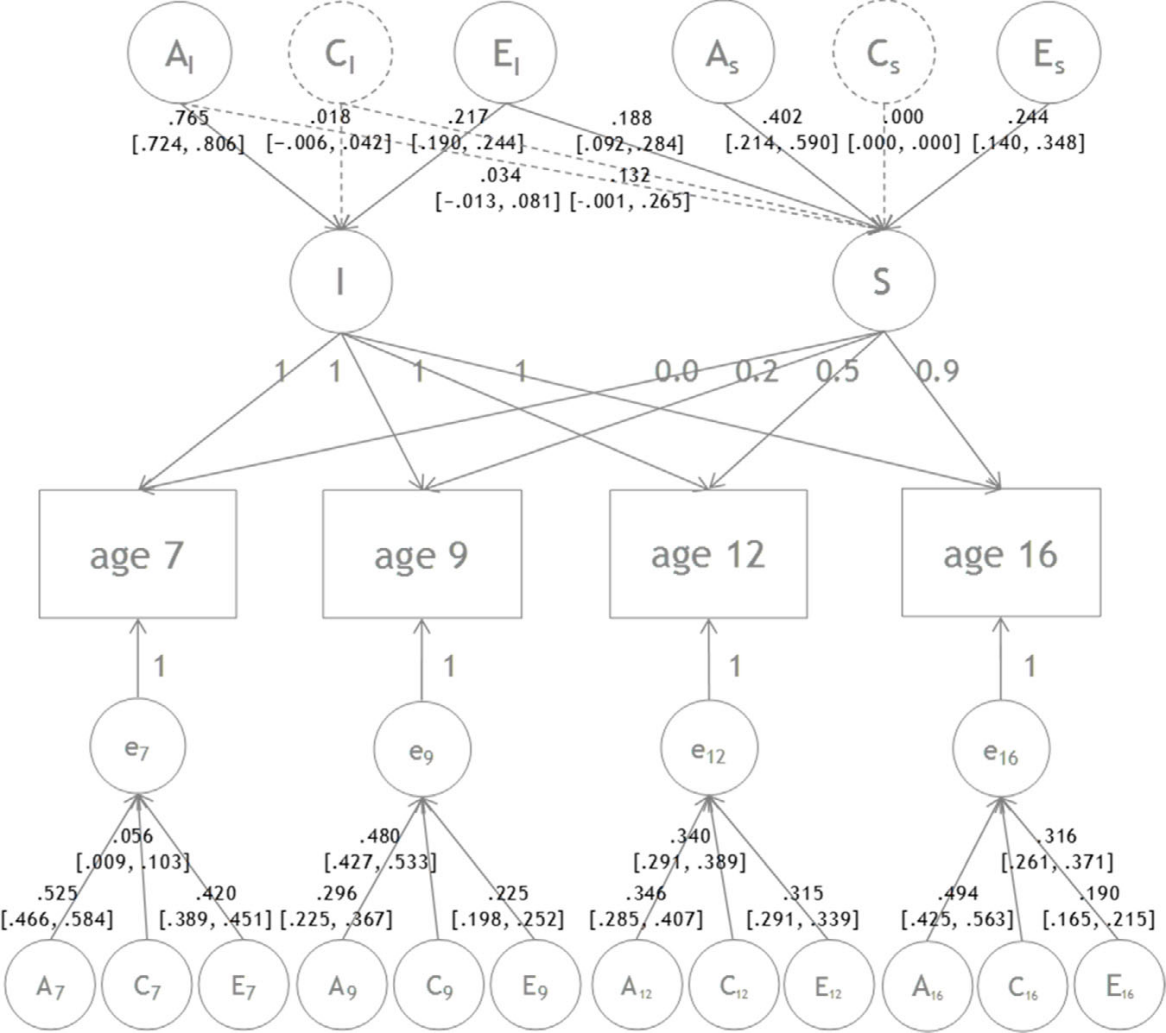
Genetic and environmental influences on the intercept and slope of CU between ages 7 and 16. The intercept (I) and the slope (S) and their loadings are indicated. A (additive genetic effect), C (shared environmental effect), E (nonshared environmental effect) standardized components of variance and 95% confidence intervals are provided for both I and S

ACE variance decomposition was conducted on linear growth model. ACE standardized variance components for the baseline level and the slope are reported in Figure 2. Heritability of the baseline level (i.e. intercept) of CU was high: 76.5% (95% CI: 72.4%−80.6%) of the variance was explained by additive genetic influences. Although, as shown in Tables 2 and 3, the univariate estimates of heritability were lower than 65% for all waves, latent factors are often found to be more heritable because measurement error is lower. It is therefore unsurprising that the heritability estimate for the intercept is larger than the univariate estimate for the initial time point (e.g. Henry et al., 2018; Pingault, Rijsdijk et al., 2015; Pingault, Viding et al., 2015). Nonsignificant shared environment component and small but significant nonshared environment components were observed [1.8% (−0.6%, 4.2%) for C, and 21.7% (19.0%, 24.4%) for E]. Individual differences in the systematic linear change (slope) of CU were moderately genetically influenced: 43.6% [25.2%, 62.0%]. Almost all of this genetic variance was due to genetic factors specific to the slope [40.2% (21.4%, 59.0%)] rather than shared with the baseline level. Moderate nonshared environmental influences were detected on the slope [43.2% (27.3%, 59.1%)]. Almost half of the nonshared environmental factors influencing the slope of CU traits overlapped with nonshared environmental factors that influenced baseline levels of CU traits [18.8% (9.2%, 28.4%) shared; 24.4% (14.0%, 34.8%) specific to the slope]. Statistically significant shared environmental influences were not detected on either the intercept or the slope despite shared environmental influences being evident in univariate genetic models at each age except at age 7, which means that shared environmental effects do not contribute to the baseline level, stability or systematic change over the whole period in this study.

The model also included ACE decomposition of the time‐specific residuals (i.e. percentages of variance at each age which were not explained by the latent growth factors). Figure 2 shows that time‐specific heritability varied between 29.6% [22.5%, 36.7%] (age 9) and 52.5% [46.6%, 58.4%] (age 7). Time‐specific shared environment significantly contributed between 5.6% [0.9%, 10.3%] (age 7) and 48.0% [42.7%, 53.3%] (age 9), and time‐specific nonshared environment with time‐specific measurement error explained between 19.0% [16.5%, 21.5%] (age 16) and 42.0% [38.9%, 45.1%] (age 7) of the variance.

## Discussion

The aim of this study was to clarify the genetic and environmental aetiology of the developmental course of CU traits between the ages of 7 and 16 years. Individual differences in the baseline level of CU traits showed high heritability (76.5%), while individual differences in the developmental course of CU traits (i.e. systematic linear change) were moderately heritable (43.6%). Furthermore, genetic influences underlying the developmental course of CU traits were largely independent from those underlying the baseline level. Small nonshared environmental contributions were detected for the baseline level of CU traits (21.7%). However, nonshared environment explained 43.2% of the variance in the developmental course of CU traits and the nonshared influences between baseline level and developmental course substantially overlapped. Although the shared environment did not significantly contribute to the baseline levels of CU traits or their developmental course, there were significant time point‐specific shared environmental influences on CU traits except at age 7.

Our findings demonstrate that rather than only being conceptualized as factors of stability, genes also play a dynamic role in explaining systematic change in CU traits. Prior person‐centred analyses by our group show that both genetic and environmental factors play a role in explaining whether individuals show stable (i.e. high or low) or changing (i.e. increasing or decreasing) levels of CU traits across childhood (Fontaine et al., 2010). This study adds to the prior evidence base by telling us the genetic effects for the initial risk and subsequent development of CU traits are not the same. This study also tells us that in addition to genetic factors, individual‐specific environmental influences play an important role in explaining why some children will increase or maintain their CU traits over time, whereas other will desist.

New insights into the aetiology of change in CU traits might be gained by integrating developmental models to future molecular genetic investigations of CU traits, as different sets of genes may influence the developmental course versus the baseline level of CU traits (i.e. age‐related genetic heterogeneity; Pingault, Rijsdijk et al., 2015; Pingault, Viding et al., 2015). Indeed, a failure to integrate developmental models can leave specific genetic variants undetected (as has been shown for physical conditions such as obesity; Lasky‐Su et al., 2008). We speculate that genetic factors influencing the baseline level of CU traits may be related to the temperamental make‐up of the child, including those genetic variants that influence emotional reactivity or drive social affiliation and resonating with other people (Bird, & Viding, 2014; Viding & McCrory, 2019). Lack of empathy, antagonism and reduced prosocial enjoyment characterize individuals with CU traits (Blair, Leibenluft, & Pine, 2014; Sherman, & Lynam, 2017; Viding & McCrory, 2019). There is also emerging evidence that infants and small children at risk of developing CU traits show atypical social orienting and resonance with others' emotions (Bedford, Wagner, Rehder, Propper, Willoughby, & Mills‐Koonce, 2017; Hoyniak, Bates, Petersen, Yang, Darcy, & Fontaine, 2018; Wagner, Mills‐Koonce, Propper, Willoughby, Rehder, Moore, & Cox, 2016). A second set of genetic factors influencing the developmental course of CU traits may relate more specifically to traits and capacities that mature in childhood and adolescence and are likely to impact upon expression of CU traits over time. As an example, the capacity to engage in complex, goal‐oriented thinking substantially increases across childhood and adolescence (Crone & Steinbeis, 2017), as does the sensitivity to what other people think (Foulkes & Blakemore, 2018). Both are thought to be linked to changes in the adolescent brain (Crone & Steinbeis, 2017; Foulkes & Blakemore, 2018). These processes may be important for assessing best strategies for executing one's own goals, which may result in less or more adaptive ways of interacting with others. Developmental changes like this could lead to genuine changes in the appreciation and understanding of others' emotions, for example, or might reflect masking or unmasking of baseline dispositional traits – as superior or inferior (compared to the age group) planning and regulatory capacities emerge.

In addition to evidence of genetic maturation being key in driving systematic change in CU traits, our findings show that nonshared environmental effects also play an important role. Two prior MZ twin differences studies have investigated the potential role of harsh/negative or warm parenting as nonshared environmental factors contributing to twin differences in MZ CU traits. A longitudinal investigation by our group, controlling for baseline differences in CU traits, did not find a nonshared environmental effect of harsh/negative parenting on the development of subsequent CU traits (Viding, Fontaine, Oliver, & Plomin, 2009). A subsequent cross‐sectional investigation did report that MZ twin differences in harsh/negative parenting and warm parenting were associated with twin differences in CU traits (Waller, Hyde, Klump, & Burt, 2018). The twin receiving more harsh/negative parenting had more CU traits, and the twin receiving more warm parenting had less CU traits. However, given the cross‐sectional design, it is not possible to rule out that these associations did not reflect pre‐existing twin differences in CU traits that the parents responded to. Despite the pervasiveness of nonshared environmental influences on psychological traits, we have not made a huge amount of progress in identifying these influences. It has been speculated that these influences may to a substantial extent be stochastic, rather than systematic, and gene–environment interplay is likely to further complicate matters (e.g. Burt, 2009; Plomin, DeFries, Knopik, & Neiderhiser, 2016). Furthermore, we do not have a wealth of rich, longitudinal data with sensitive measurement of environment – including consideration of age‐appropriate, candidate environmental variables. This area warrants more attention from researchers.

Of interest is the role of shared environment in the present study. We detected small‐to‐substantial shared environmental influences on time‐specific residuals (i.e. the variance not explained by the baseline level and the developmental course). However, shared environmental risk factors did not contribute to the developmental course of CU traits. Although it is common to find that shared environment makes only a small contribution to child temperament and personality traits, it is consistently detected in relation to most dimensions of child and adolescent psychopathology (Burt, 2009) and has been shown to also contribute to the stability of many psychopathological symptom dimensions, including conduct problems (Pingault, Viding et al., 2015; Viding & Kimonis, 2018). The lack of significant shared environmental effects on the stability and systematic change in CU traits throughout the period is perhaps not surprising as factors in the environment that contribute to make twins similar in childhood (e.g. family, school and neighbourhood) may give way to increasingly individual‐specific influences as they grow older. However, combined results from the Cholesky decomposition (i.e. stability paths from 7 to 9 years and from 9 to 12 years) and the latent growth model (i.e. time‐specific residuals) did indicate that there were shared environmental effects at several measurement points from childhood to adolescence, suggesting that it may contribute to relatively short‐term stability over one or two ages but not contribute to systematic stability and change over the whole period.

### Limitations

A number of limitations should be noted when interpreting these findings. First, although based on well‐validated items used in prior research, our seven‐item CU trait measure showed acceptable (rather than high) internal consistency. Lower internal consistency may inflate *e* ^2^, which includes nonshared environmental influences and measurement error. The model we used partly accounted for this by modelling latent factors (less susceptible to measurement error) as well as time‐specific residual variance (capturing more error). Furthermore, the *e* ^2^ estimates for latent factors (i.e. intercept and slope) in our study were lower than the estimates reported in research using observed measures of CU traits. Second, the participants were drawn from a population‐based sample of twins from England and Wales. Replications are needed with participants from more diverse backgrounds to verify the generalizability of the findings. Finally, although we focused on a longer time span than prior genetically informative studies, we were not able to cover a period from early childhood to early adulthood. Future studies focusing on a longer time span and more measurement points will be able to further elucidate the aetiology of development and change in CU traits.

## Conclusions

In a large sample of twins with repeated measures of CU traits throughout childhood and adolescence, we showed that individual differences in the baseline level and the systematic change in CU traits were under substantial and nonoverlapping genetic influences. Such differences in genetic influences across ages may make genomewide association studies of CU traits challenging, as heterogeneity in age within or between samples may undermine the detection of associations and replication attempts. New genetic and environmental influences with age suggest that repeated and age‐tailored interventions may be required throughout development to make a lasting difference in the presentation of CU traits and associated outcomes.

## Supporting information

**Appendix S1.** Details of zygosity determination.
**Appendix S2.** Justification of the CU trait measurement.
**Table S1.** Fit indices for confirmatory factor analyses.
**Table S2.** Loadings on the single factor of CU traits for all ages by the confirmatory factor analyses.
**Table S3.** Tucker's congruence coefficients (in the upper triangular matrix) and factorial correlations (in the lower triangular matrix).
