# Supplementary material for: Genetic and environmental influences on the developmental trajectory of callous‐unemotional traits from childhood to adolescence
Source: J Child Psychol Psychiatry. 2020 May 17;62(4):414–23. doi: 10.1111/jcpp.13259 (PMC8432158; doi:10.1111/jcpp.13259)
Supplement: Supplementary file 1 — Appendix S1. Details of zygosity determination. Appendix S2. Justification of the CU trait measurement. Table S1. Fit indices for confirmatory factor analyses. Table S2. Loadings on the single factor of CU traits for all ages by the confirmatory factor analyses. Table S3. Tucker's congruence coefficients (in the upper triangular matrix) and factorial correlations (in the lower triangular matrix). [file JCPP-62-414-s001.docx]

**Supporting information – Genetic and environmental influences on the developmental trajectory of Callous-Unemotional traits from childhood to adolescence – by Takahashi *et al*.**

**Appendix S1.** Details of zygosity determination.

Zygosity was assessed using a parent questionnaire of physical similarity, which is approximately 95% accurate when compared with DNA testing (Price, Freeman, Craig, Petrill, Ebersole, & Plomin, 2000). DNA testing was conducted when zygosity was not clear from the physical similarity criteria.

**References:**

Price, T.S., Freeman, B., Craig, I., Petrill, S.A., Ebersole, L., & Plomin, R. (2000). Infant zygosity can be assigned by parental report questionnaire data. *Twin Research*, *3*, 129 –133.

**Appendix S2.** Justification of the CU traits measurement.

A number of past studies have measured individual differences in CU traits with 7 or 9 items from both APSD and SDQ (e.g. Dadds, Fraser, Frost, & Hawes, 2005; Viding et al., 2005, 2007; Moul, Dobson-Stone, Brennan, Hawes, & Dadds, 2013). These items have shown good internal consistency, capture unique variance and capture a distinct subgroup of children with conduct problems.

To ensure that the 7-item measure used in this study provided appropriate measurement of CU traits a series of confirmatory factor analyses (CFA) was conducted to confirm CU traits at each assessment point could be construed as a single factor. As shown in Table S1, in terms of several model fit indices, we could confirm that a single factor structure of the CU traits measure used in this study fit the data well for all age groups. Moreover, measurement invariance was tested across different assessment points (i.e. different ages). As seen in Table S1, there was weak invariance over time, which indicates that we can assume the same single factor structure with the same factor loadings over time. No strong invariance could be verified. Lastly, a four-factor (corresponding to the four ages) correlated CFA model fit the data well (*χ*2 = 1691.051, *df* = 302, *p* < .01, CFI = .935, RESEA = .023, and SRMR = .032). Factor loadings are shown in Table S2. Additionally, to evaluate factorial invariance, the Tucker’s congruence coefficients for each of the age groups across the two matrices were computed as the cross products of two column vectors of normalized factor loadings of interest (Haven & ten Berge, 1977). Congruence coefficients among the age groups are presented in the upper triangular matrix in Table S3. Factors loadings were highly congruent, with congruence coefficients ranging from .975 to .993. These results suggest factorial invariance because conventional criteria with coefficients above .90 are taken as evidence of factor replication (Barrett, 1986). Last, to evaluate the stability over time, inter-factorial correlations were simply calculated with the abovementioned four-factor correlated CFA model. As presented in the lower triangular matrix in Table S3, factorial correlations among the age groups were moderate, ranging from .460 for ages 7 to 16 to .699 for ages 9 to 12, which shows a level of phenotypic continuity across ages well in line with other behavioural studies in childhood and adolescence. Taken together, the items used in this study were developmentally-invariant and single-factor measure to assess individual differences in CU traits.

**References:**

Barrett, P. (1986). Factor comparison: An example of three methods. Personality and Individual Differences, 7, 327–340.

Dadds, M.R., Fraser, J., Frost, A., & Hawes, D.J. (2005). Disentangling the underlying dimensions of psychopathy and conduct problems in childhood: A community study. *Journal of Consulting and Clinical Psychology*, *73*, 400–410.

Feldstein Ewing, S.W., & Luciana, M. (Eds.). (2018). The Adolescent Brain Cognitive Development (ABCD) Consortium: Rationale, aims, and assessment strategy [Special issue]. *Developmental Cognitive Neuroscience*, *32*, 1−164.

Haven, S., & ten Berge, J.M.F. (1977). Tucker’s coefficient of congruence as a measure of factor invariance: An empirical study. *Heymans Bulletin*, 290EX. Groningen, the Netherlands: University of Groningen.

Hawes, S.W., Waller, R., Thompson, W.K., Hyde, L.W., Byrd, A.L., Burt, S.A., ... & Gonzalez, R. (in press). Assessing callous-unemotional traits: development of a brief, reliable measure in a large and diverse sample of preadolescent youth. *Psychological Medicine*.

Moul, C., Dobson-Stone, C., Brennan, J., Hawes, D., & Dadds, M. (2013). An exploration of the serotonin system in antisocial boys with high levels of callous-unemotional traits. *PLoS ONE*, *8(2)*: e56619.

**Table S1.** Fit indices for confirmatory factor analyses.

| Models | *χ*^2^ | *df* | *p* | CFI | RMSEA | SRMR | Δ*χ*^2^_(d)_ | Δ*df* | *p* | ΔCFI | ΔRMSEA | ΔSRMR | Model Comparison |
| --- | --- | --- | --- | --- | --- | --- | --- | --- | --- | --- | --- | --- | --- |
| Each age group |  |  |  |  |  |  |  |  |  |  |  |  |  |
| age 7 | 373.665 | 14 | < .01 | .904 | .058 | .037 |  |  |  |  |  |  |  |
| age 9 | 290.680 | 14 | < .01 | .867 | .077 | .048 |  |  |  |  |  |  |  |
| age 12 | 329.114 | 14 | < .01 | .900 | .063 | .038 |  |  |  |  |  |  |  |
| age 16 | 352.508 | 14 | < .01 | .936 | .070 | .039 |  |  |  |  |  |  |  |
| Multigroup |  |  |  |  |  |  |  |  |  |  |  |  |  |
| Configural invariance | 1357.892 | 56 | < .01 | .910 | .065 | .035 | ̶ | ̶ | ̶ | ̶ | ̶ | ̶ | ̶ |
| Weak invariance | 1676.313 | 77 | < .01 | .889 | .061 | .050 | 318.420 | 21 | < .01 | .021 | −.004 | .015 | Weak vs. Configual |
| Strong invariance | 3429.201 | 95 | < .01 | .768 | .080 | .068 | 1752.890 | 18 | < .01 | .121 | .019 | .018 | Strong vs. Weak |

**Table S2.** Loadings on the single factor of CU traits for all ages by the confirmatory factor analyses.

|  | factor loadings | | | |
| --- | --- | --- | --- | --- |
|  | age 7 | age 9 | age 12 | age 16 |
| 1. Does not show feelings or emotions | .074 | .159 | .204 | .263 |
| 2. Helpful if someone is hurt, upset or feeling ill (R) | .622 | .706 | .635 | .714 |
| 3. Feels bad or guilty when he/she does something wrong (R) | .367 | .382 | .363 | .461 |
| 4. Has at least one good friend (R) | .195 | .154 | .247 | .320 |
| 5. Considerate of other people's feelings (R) | .603 | .645 | .638 | .751 |
| 6. Kind to younger children (R) | .521 | .504 | .465 | .538 |
| 7. Is concerned about how well he/she does at school (R) | .174 | .238 | .229 | .389 |

*Note*. R: reversed items.

**Table S3.** Tucker’s congruence coefficients (in the upper triangular matrix) and factorial correlations (in the lower triangular matrix).

|  | age 7 | age 9 | age 12 | age 16 |
| --- | --- | --- | --- | --- |
| age 7 | − | .993 | .989 | .975 |
| age 9 | .689 | − | .993 | .983 |
| age 12 | .589 | .699 | − | .991 |
| age 16 | .460 | .513 | .606 | − |
